# Characterization and phylogenetic significance of the complete chloroplast genome of *Camellia Kissii*, an economic crop for producing oil

**DOI:** 10.1080/23802359.2019.1703569

**Published:** 2020-01-07

**Authors:** Lei Cao, Jiyuan Li, Zhengqi Fan, Hengfu Yin, Xinlei Li

**Affiliations:** a College of Information Science and Technology, Nanjing Forestry University, Nanjing, China;; b State Key Laboratory of Tree Genetics and Breeding, Research Institute of Subtropical, Forestry, Chinese Academy of Forestry, Hangzhou, China;; c Key Laboratory of Forest Genetics and Breeding, Research Institute of Subtropical, Forestry, Chinese Academy of Forestry, Hangzhou, China

**Keywords:** *Camellia kissii*, chloroplast genome, oil production, phylogenetic significance

## Abstract

*Camellia kissii* is cultivated for a long time as an oil crop for edible and industrial oils, and has the functions of high oil production rate and unique health care. The complete chloroplast (cp) genome sequence of *C. kissii* is 156,961 bp in length with GC content of 39.29%. It presents a quadrate structure, including a large single-copy (LSC) region (86,640 bp), a small single-copy (SSC) region (18,399 bp), and a pair of inverted repeats (IRs) (25,961 bp). Meanwhile, 15 complete chloroplast genome of *Camellia* was aligned to explore the phylogenetic significance of *Camellia*. And the genetic relationship between *Camellia kissii* and *Camellia huana* was found to be closest.

*Camellia kissii*, which grows in the evergreen broad-leaved forest, is a kind of crop with important economic value. At present, *C. kissii* is cultivated for a long time as an oil crop for edible and industrial oils, and has the functions of high oil production rate and unique health care (Cao et al. 2016). However, the genomic information of *C. kissii* and the genetic information between the populations are vacant. In this paper, we report the complete chloroplast genome of *C. kissii* and evaluate the phylogenetic relationships among its associated populations, which accelerates the expansion of *Camellia* resources.

The samples of *C. kissii* were collected from Jinhua International Camellia Species Park (Zhejiang, China; Coordinates: 29°7′10.1208″N, 119°35′52.1088″E). Illumina Hiseq platform, 2 × 150 two-terminal sequencing strategy was used to sequence the library. The complete chloroplast genome was assembled using a comparative mapping method (Bi et al. 2016, Wang et al. 2018), and its native *Camellia japonica* (NCBI accession Number: NC_036830.1) was selected as the initial reference genome. The annotated chloroplast genome of *Camellia kissii* has been submitted to GenBank (NCBI Accession Number MN635793). The voucher specimen (CD_02) was reserved in State Key Laboratory of Tree Genetics and Breeding, Research Institute of Subtropical Forestry, Chinese Academy of Forestry.

During the process of genome assembly, we have carried out strict control and quality evaluation of sequence data. After filtering 712,687 raw reads (161,737,760 raw bases), 28,023,651 clean reads (4,017,525,007 raw bases) were aligned to *Camellia japonica* through the Bowtie2 software (Langmead and Salzberg 2012). Then, 654,723 unduplicated sequences were aligned to the reference genome, reaching 30.36 coverage. The sequence of cp genome was assembled using Newbler v3.0 (Ye et al. 2017) with the default parameters. Finally, the complete cp genome sequence of *C. kissii* is 156961 bp in length with GC content of 39.29%, including a large single-copy (LSC) region (86,640 bp), a small single-copy (SSC) region (18,399 bp), and a pair of inverted repeats (IRs) (25,961 bp). In addition, the GC content of LSC and SSC regions (35.29% and 30.53%) is lower than that of IR regions (42.02%). The cp genome was annotated with 129 functional genes, consisting of 81 protein-coding genes, 44 transporter RNAs, and 4 ribosomal RNAs.

In order to explore the phylogenetic evolution of *Camellia*, the entire genome of 15 chloroplasts of *Camellia* was aligned using MEGA v7.0.14 (Kumar et al. 2016). Neighborhood addition method (Saitou and Nei 1987) is used to infer its evolutionary history. The percentage of replication trees (1500 replicates) in which the relevant taxonomic units are clustered in the boot test is shown next to the branch (Figure 1) (Felsenstein 1985). The genetic relationship between *Camellia kissii* and *Camellia huana* (KY626040.1) was found to be closest.

**Figure 1.**
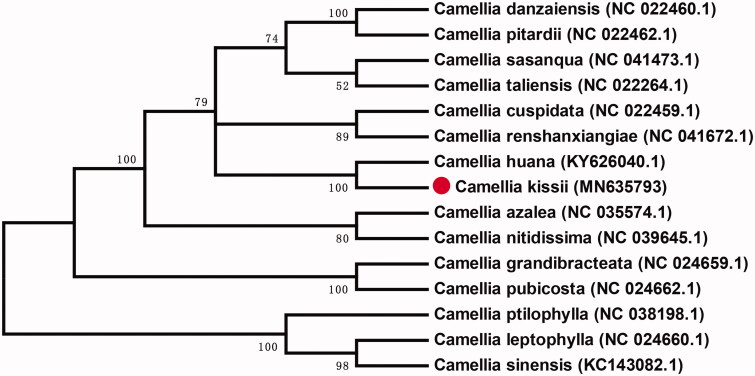
The Neighbor-joining (NJ) phylogenetic tree for *Camellia kissii* with other *Camellia* species based on conserved 75 protein sequences of cp genomes.
